# Sex- and gender-specific differences in treatment efficacy and safety of traditional Chinese medicine: a scoping review

**DOI:** 10.3389/fgwh.2026.1833361

**Published:** 2026-07-13

**Authors:** Jing Hu, Boyang Liu, Haiyin Hu, Xiaolei Wu, Paola Matarrese, Massimo D'Archivio, Anna Ruggieri, Elena Ortona, Lin Miao, Junhua Zhang, Alice Josephine Fauci, Zhaochen Ji

**Affiliations:** 1 Tianjin University of Traditional Chinese Medicine, Tianjin, China; 2 Haihe Laboratory of Modern Chinese Medicine, Tianjin, China; 3 Key Laboratory of Evidence-Based Evaluation of Traditional Chinese Medicine, National Medical Products Administration, Tianjin, China; 4 Centre for Gender-Specific Medicine, Italian National Institute of Health, Rome, Italy; 5 Sino-Italian Joint Laboratory of Traditional Chinese Medicine, Italian National Institute of Health, Rome, Italy

**Keywords:** scoping review, sex and gender medicine, sex and gender sensitive medicine, sex- and gender-specific differences, traditional Chinese medicine

## Abstract

**Background:**

While Western medicine is increasingly investigating sex- and gender-specific differences in health and disease, and despite TCM theory and practice placing particular emphasis on individual differences—including sex- and gender-related characteristics, research on sex- and gender- sensitive medicine in TCM remains underexplored.

**Objective:**

We conducted a scoping review to map and synthesize the existing evidence on sex- and gender-specific differences in the efficacy and safety of TCM interventions and to analyze possible underlying mechanisms.

**Methods:**

The scoping review followed the JBI guidance. Six Chinese and English databases (CNKI, WF, VIP, SinoMed, PubMed, and Web of Science) were searched from inception to July 2025 for preclinical and clinical studies. Key information was extracted from the included studies, and data were synthesized using descriptive statistics and systematic narrative synthesis. GraphPad Prism 10.0 was used to generate figures.

**Results:**

Database searches identified 6,720 records; after removing 250 duplicates, 6,470 records were screened by title and abstract, and 2,417 proceeded to full-text eligibility assessment. Ultimately, 16 studies were included, comprising randomized controlled trials, animal experiments, non-randomized controlled trials, and single-arm trials. Conclusions drawn from the original studies indicated that changes in all relevant outcome measures were associated with sex. Sex-specific differences in TCM efficacy were observed across herbal, acupuncture, and combined interventions. Female animals demonstrated predominant benefits in bone metabolism, whereas female human participants showed greater benefits in pain perception, emotional regulation, neurological recovery, and pulmonary functional outcomes. Male animals exhibited more pronounced gut microbiota responses to metabolic interventions, while male human participants showed stronger effects in obesity-related outcomes and sensorimotor responses. These divergent patterns likely reflect sex-related differences in pharmacokinetics, gut-brain axis interactions, and neuroendocrine mechanisms. None of the included studies examined gender-related sociocultural factors.

**Conclusion:**

This review highlights a persistent theoretical and methodological gap in TCM research, which has yet to fully integrate sex- and gender-sensitive medicine principles. The limited high-quality clinical evidence underscores the need for rigorous sex-informed trials to evaluate efficacy across sexes. These findings support the integration of sex as a key determinant in TCM clinical decision-making, alongside syndrome differentiation, to advance precision medicine and improve personalized therapeutic outcomes.

**Systematic Review Registration:**

DOI: 10.17605/OSF.IO/QABTY.

## Key messages

1) Analysis of 16 studies indicates sex-related differences in the efficacy of TCM interventions: female animals demonstrated predominant benefits in bone metabolism, whereas female human participants showed greater benefits in pain perception, emotional regulation, neurological recovery, and pulmonary functional outcomes. Male animals exhibited more pronounced gut microbiota responses to metabolic interventions, while male human participants showed stronger effects in obesity-related outcomes and sensorimotor responses. These divergent patterns likely reflect sex-related differences in pharmacokinetics, gut-brain axis interactions, and neuroendocrine mechanisms.
2) Acupuncture demonstrates greater pain relief effects in female participants (e.g., stimulation of the GB34 acupoint), while metabolic improvements are more pronounced in male participants; similarly, herbal formulations such as Yougui Pill appear to enhance bone density more effectively in female animals.
3) All included studies focused exclusively on biological sex differences, with no consideration of gender-related sociocultural factors or gender identity, highlighting a major gap in TCM research that may limit a comprehensive understanding of treatment outcomes and patient experiences.
4) Sex-related differences in TCM efficacy are driven by an integrated interplay of hormonal regulation, gut microbiota composition, and nervous system function, which collectively influence drug metabolism, target sensitivity, and therapeutic response.
5) This study highlights the need for modern research in TCM to explicitly consider the biological relevance of sex- and gender-related factors, and to integrate these perspectives into both basic research—through sex-equitable research strategies—and the clinical practice of personalized diagnosis and treatment.

## 1 Introduction

Sex and gender are increasingly recognized as important determinants of health and disease. Sex refers to biological attributes—including chromosomes, gene expression, hormone levels, and reproductive anatomy—while gender refers to socially constructed roles, behaviours, expressions, and identities (1). Both are multidimensional: sex exists along a continuum rather than a strict binary, and gender encompasses identity, expression, roles, and relational dimensions that interact dynamically across the lifespan (1, 2). Sex- and gender-sensitive medicine recognizes and investigates the impact of both sex-related biological characteristics and gender-related sociocultural factors on health and disease in male and female participants. Biological sex can influence gendered experiences of health and illness, while sociocultural gender norms and behaviours can in turn modify biological pathways and disease expression through mechanisms such as stress physiology, healthcare-seeking patterns, and differential exposure to environmental or occupational risks. Differences observed between male participants and female participants in physiology and pathology are not only attributable to biological sex, but also influenced by gender, which permeates the disease trajectory—prevention, diagnosis, treatment, and prognosis—though not uniformly at each stage (3–8). Growing recognition of the importance of sex and gender in healthcare (9), together with clinical experience in sex- and gender-specific interventions (10–12), has established the integration of sex- and gender-based perspectives as one of the cornerstones of contemporary precision medicine (13–15). Molecular mechanisms underlying disease differences primarily reflect biological sex (pharmacokinetics, hormone-receptor interactions, genetic expression). However, gender sensitivity remains critical to ensuring that molecular insights translate equitably into clinical practice, accounting for how sociocultural gender norms may modify access to, experiences of, and outcomes from molecularly targeted therapies. Although other social identities (such as age, ethnicity, and socioeconomic status) are widely recognized as influencing health and disease, their complex, multidimensional intersectional effects remain difficult to fully disentangle and evaluate. Sex and gender, while remaining subjects of ongoing conceptual and academic discussion, represent important and increasingly operationalizable dimensions in biomedical research and healthcare. Sex, as a biological variable, influences virtually every organ system and cellular process, while gender, as a sociocultural construct, shapes health behaviours, exposures, and interactions with healthcare systems throughout the lifespan. Their broad and well-documented impact on health outcomes positions “sex- and gender-sensitive medicine” as a foundational pillar of precision medicine.

Traditional Chinese Medicine (TCM) is a codified system of traditional medicine originating in China, with a theoretical framework encompassing Yin-Yang, the Five Elements, Zang-Xiang (visceral manifestations), Qi-Blood-Body Fluids, and meridians, and diagnostic methods including the four diagnostic methods (observation, auscultation-olfaction, inquiry, palpation) and pattern differentiation (bian zheng) (15). In 2019, the World Health Organization (WHO) included traditional medicine conditions in ICD-11 (Supplementary Chapter 26), enabling standardized counting and international comparison of traditional medicine services (16). TCM encompasses diverse therapeutic modalities, including herbal medicine, acupuncture, moxibustion, massage, qigong, and dietary therapy. TCM's emphasis on the individualized care conceptually aligns with “sex and gender medicine” principles: core theories, including syndrome differentiation and constitution theory, account for individual variability shaped by age, environment, and sex (17, 18). In classical theory, rooted in the Huangdi Neijing (The Yellow Emperor's Inner Canon), sex differences are explicitly acknowledged within the Yin-Yang framework, with distinct physiological characteristics assigned to man (yang-dominant, qi-abundant) and woman (yin-dominant, blood-abundant). However, these traditional considerations are limited to the biological (sex) dimension; classical TCM theory does not address gender as a sociocultural construct. Several questions, therefore, remain unanswered: which TCM interventions have been evaluated through a sex- and gender-sensitive lens; which health conditions have been investigated; what outcome measures and mechanisms have been explored; and where evidence gaps persist. Addressing these questions is essential for integrating sex- and gender-specific considerations into TCM research and for aligning TCM practice with contemporary precision medicine.

This scoping review aims to systematically map and synthesize existing evidence on sex- and gender-specific differences in the efficacy and safety of TCM interventions, and to discuss potential mechanisms from both biomedical and TCM perspectives.

A scoping review methodology was selected to capture the breadth of existing evidence and identify research priorities. A review protocol was prospectively registered prior to the initiation of this scoping review. The registration is publicly accessible via the DOI: 10.17605/OSF.IO/QABTY.

## 2 Methods

### 2.1 Study design

This scoping review was conducted in accordance with the Johanna Briggs Institute (JBI) (19, 20) methodology for scoping reviews and is reported following the PRISMA extension for scoping reviews (PRISMA-ScR) (21). The research question “What evidence exists on sex- and gender-specific differences in TCM efficacy and safety of Traditional Chinese Medicine interventions?” was formulated using the Population, Concept, and Context (PCC) framework. The PCC framework is presented in Table 1.

**Table 1:** Pcc framework.

PCC Element	Application in this Review
P—Population	Human participants of any age or health status, animal models and *in vitro* biological systems (primary cells, cell lines) used in TCM research
C—Concept	Sex- and gender-specific differences in the efficacy, mechanisms, pharmacokinetics, pharmacodynamics, or safety of Traditional Chinese Medicine (TCM) interventions
C—Context	All settings and study designs, including clinical and preclinical studies; no restrictions on geography or publication year

### 2.2 Eligibility criteria

We included preclinical and clinical studies that investigated TCM interventions and reported sex- or gender-related differences in efficacy, safety, pharmacokinetics, pharmacodynamics, or underlying mechanisms. In this review, sex-related differences refer to differences in outcomes between male and female participants (or male and female animals in animal experiments) (35), where sex was classified based on self-report, clinical records, or biological markers as reported in the original studies, whereas gender referred to self-reported sociocultural identity and related constructs when assessed by the study authors. In preclinical (animal) studies, sex was defined based on biological characteristics, including chromosomal and hormonal features. The specific gender dimension examined was identified during data extraction based on the original study reports. Eligible interventions included herbal medicines (single herbs, extracts, or compound formulations), acupuncture and related techniques (e.g., electroacupuncture, moxibustion, acupressure), and other non-pharmacological TCM therapies (e.g., Daoyin, Qigong, Tui Na). Studies were eligible if they directly compared outcomes between male and female participants (or male and female animals in animal experiments) or provided sex-disaggregated data enabling sex-specific analyses. No restrictions were applied regarding study design, setting, geographical location, or publication year. Only studies published in Chinese or English were included.

We excluded: (1) studies that did not involve TCM interventions; (2) studies that neither reported “sex- or gender-related” factors nor “sex- or gender-disaggregated” data; (3) publications lacking empirical data (e.g., editorials, commentaries, opinion pieces, narrative reviews); (4) conference abstracts and grey literature; (5) studies published in languages other than Chinese or English; (6) articles with unavailable full-text; (7) duplicates.

### 2.3 Search strategy

A comprehensive literature search was performed in four Chinese databases (CNKI, WanFang, VIP, and SinoMed) and two English-language databases (PubMed and Web of Science) from database inception to July 2025. Search strategies were developed in three steps, following the JBI Manual for Evidence Synthesis (21). An initial limited search was conducted in PubMed to identify keywords and thesaurus terms related to the topics. The second step involved formulating a search strategy with all the keywords identified and conducting a comprehensive search across all included databases, adapted according to the specific characteristics of each database. Search strategies for each database are detailed in supplementary Appendix 1. The third step involved searching the reference lists of the included studies and screening relevant review articles to identify additional eligible studies (a secondary reference screening). No further studies meeting the inclusion criteria were identified through this process.

### 2.4 Screening and data extraction

All retrieved records were imported into reference management software (NoteExpress 3.0), and duplicates were removed. Two reviewers (Jing HU, Haiyin HU) independently screened titles and abstracts, followed by full-text assessment against the predefined eligibility criteria. Disagreements were resolved through discussion or consultation with a third reviewer (Junhua ZHANG). The study selection process is presented in a PRISMA 2020 flow diagram (36).

The data were extracted using a standardized charting form that was independently developed by one reviewer (Zhaochen JI). Extracted variables included: bibliographic data (title, author, publication year, journal); study characteristics (design, sample size, population type, setting); intervention details (TCM modality, dosage, duration, controls); outcome measures and methods (efficacy, safety, mechanism indicators); sex- and gender-related findings (quantitative differences, statistical significance). Data extraction was conducted independently by two reviewers (Jing HU, Boyang LIU), with discrepancies resolved through discussion or consultation with a third reviewer (Alice Josephine Fauci).

### 2.5 Critical appraisal of sources of evidence

Although it is not required for scoping reviews, a formal assessment of the methodological quality of included studies was conducted to contextualize the strength and limitations of the available evidence. Methodological quality did not constitute an exclusion criterion (37).

Randomized controlled trials were evaluated using the Cochrane Risk of Bias tool; non-randomized studies were assessed using the Methodological Index for Non-Randomized Studies (MINORS) or the Newcastle–Ottawa Scale, as appropriate; and animal studies were appraised using relevant reporting and bias assessment frameworks (38–41).

### 2.6 Data synthesis

Given the heterogeneity of study designs, interventions, and outcome measures, a quantitative meta-analysis was not performed. Findings were synthesized descriptively, with emphasis on identifying patterns of sex- and gender-specific differences across intervention types and outcome domains. Results are presented through narrative synthesis and visual summaries to facilitate comparison across studies.

## 3 Results

**Figure 1:**
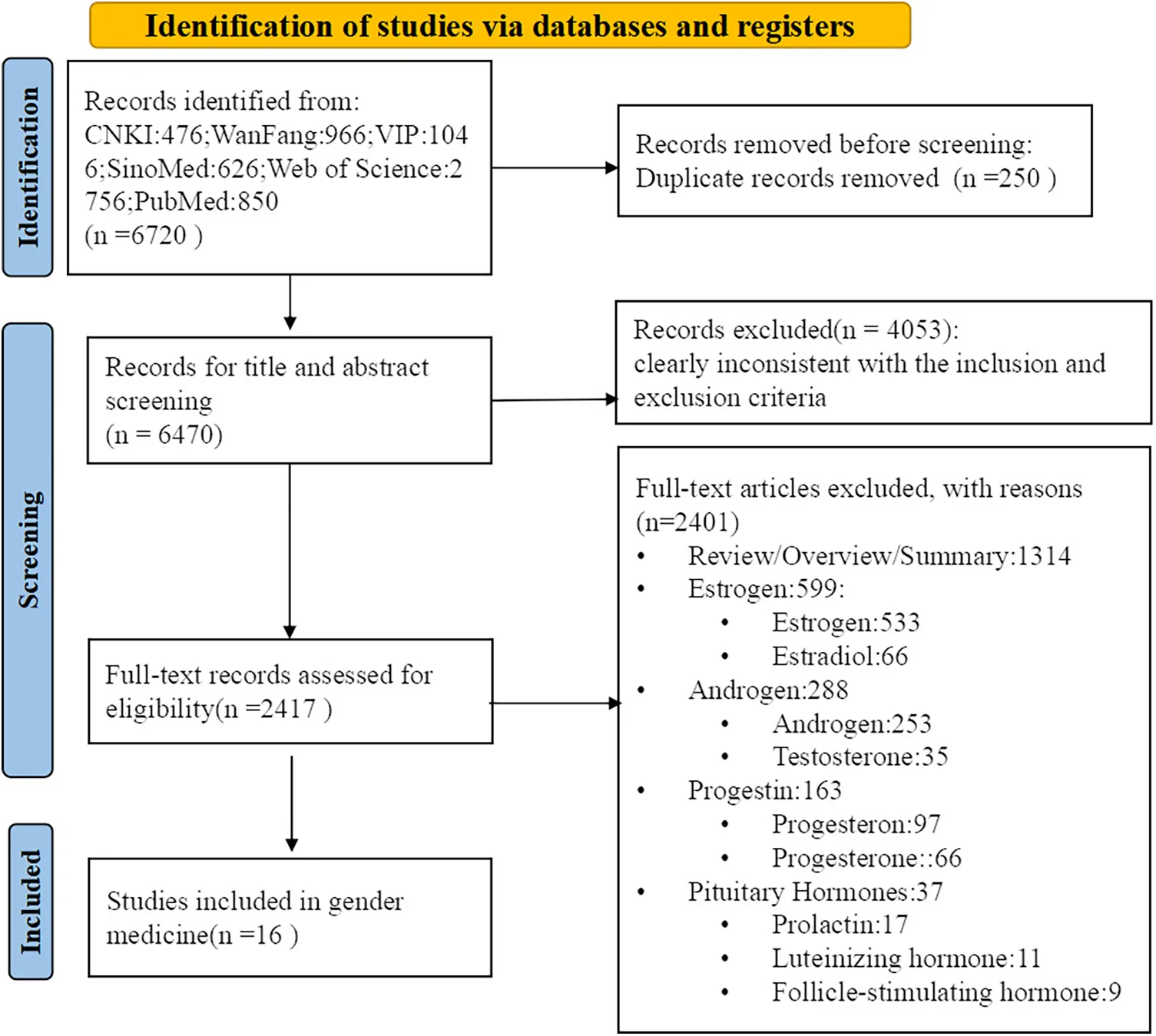
PRISMA 2020 flow diagram of the study selection process.

The literature search yielded a total of 6,720 records across all databases. After removal of duplicates, 6,470 records were screened by title and abstract; 4,053 studies were excluded at this stage for failing to meet the criteria, leaving 2,417 studies eligible for full-text evaluation. During the full-text review, 2,401 additional studies were excluded based on two specific reasons: (1) the study design was neither a clinical trial nor an experimental study (*n* = 1,314, 54.4%); (2) the research focused solely on sex hormones or pituitary hormones with no relevant data on TCM interventions (*n* = 1,087, 45.0%). Ultimately, 16 studies met all the inclusion criteria and were included in the final scoping review. The selection process is illustrated in Figure 1.

### 3.1 Characteristics of included studies

The included studies comprised both preclinical and clinical research investigating sex-related differences in response to TCM interventions. Overall, 5 studies were conducted in animal models, while 11 were clinical trials involving human participants: randomized controlled trials (RCTs, *n* = 5, 31%), non-randomized controlled trials (CCTs, *n* = 4, 25%), and single-arm trials (SAs, *n* = 2, 13%). No *in vitro* studies addressing sex-related or gender-related differences in TCM interventions were identified in our analysis. A summary of the included studies' characteristics is presented in Table 2.

**Table 2:** General characteristics of the included studies.

Article	Country	Types	Comparison Type	Core Focus	Tools	Quality	Limitations
Chang Shik Yin (22)	Korea	SA	Inter-group	Acupuncture for allergic rhinitis (sex-based response)	MINORS	Good (12/16, 75%)	Endpoint objectivity not reported (⑤=0); no sample size estimation (⑧=0)
Weiqiao Qiu 2009	USA	CCT	Inter-group	Acupuncture for chemotherapy-induced fatigue (gender differences)	MINORS	Good (18/24, 75%)	Endpoint objectivity not reported (⑤=0); insufficient follow-up (⑥=0); no sample size estimation (⑧=0)
Branden A. Smeester (23)	USA	AE	Inter-group	Electroacupuncture for hypertension (sex-specific efficacy)	SYRCLE	Some concerns	High risk in sequence generation; allocation concealment and blinding unclear
Haiyan Xu (24)	China	AE	Inter-group	Ginseng extract for vascular dysfunction (gender-dependent mechanism)	SYRCLE	Some concerns	High risk in sequence generation; multiple domains with unclear risk including allocation, blinding, and baseline adjustment
Xiaowei Fu 2014	China	AE	Inter-group (subgroup)	Herb pair for osteoporosis (sex differences in bone metabolism)	SYRCLE	Moderate	No high-risk items; allocation concealment, baseline adjustment, blinding, and outcome assessment unclear
Ling Fan (25)	China	RCT	Intra-group	Acupuncture for depression (gender-related efficacy)	Cochrane RoB2	Low	Allocation concealment not reported; blinding of outcome assessment unclear
Sujung Yeo (26)	Korea	CCT	Inter-group	Acupuncture for menopause (sex hormone differences)	MINORS	Good (20/24, 83%)	Endpoint objectivity not reported (⑤=0); no sample size estimation (⑧=0)
Natasha L. Fabiaña (27)	Singapore	RCT	Intra-group	Acupuncture for post-stroke fatigue (gender-based effect)	Cochrane RoB2	Low	Identified as short communication; limited methodological detail
Didi Huang 2018	China	CCT	Inter-group	Moxibustion for constipation (sex differences in clinical efficacy)	MINORS	Good (20/24, 83%)	Endpoint objectivity not reported (⑤=0); no sample size estimation (⑧=0)
Anupama Kizhakkeveettil MAOM (28)	USA	RCT	Intra-group	Acupuncture for migraine (gender-specific response)	Cochrane RoB2	High	High risk in measurement of outcomes; insufficient blinding; selective reporting concerns
Yanan Ding (29)	China	AE	Intra-group	Herb formula for rheumatoid arthritis (sex-dependent mechanism)	SYRCLE	Some concerns	High risk in random outcome evaluation; allocation concealment and blinding unclear
Huihui Xu (30)	China	AE	Inter-group (subgroup)	Chinese herb for insulin resistance (gender-based efficacy)	SYRCLE	Moderate	No high-risk items; allocation concealment, blinding, outcome assessment, and other bias unclear
Yanping Zhao (31)	China	SA	Inter-group	Acupuncture for insomnia (gender differences in brain response)	MINORS	Moderate (10/16, 63%)	Endpoint objectivity not reported (⑤=0); loss to follow-up >5% (⑦=0); no sample size estimation (⑧=0)
Saiqin Hu (32)	China	CCT	Inter-group	Moxibustion for hypertension (sex-specific effect)	MINORS	Good (12/16, 75%)	Endpoint objectivity not reported (⑤=0); no sample size estimation (⑧=0); assessed with non-comparative items only
Di Miao (33)	China	RCT	Inter-group	Herb formula for acne (gender-based efficacy)	Cochrane RoB2	High	High risk in outcome measurement and selective reporting; no blinding of outcome assessors
Chi Hou (34)	China	RCT	Intra-group	Acupuncture for COVID-19 (sex differences in symptom relief)	Cochrane RoB2	Low	Subgroup reanalysis design; allocation concealment and blinding adequately reported

RCT, randomized controlled trial; CCT, non-randomized controlled clinical trial; SA, single-arm trial; AE, animal experiment.

**RoB2 domains:** D1, Randomization process; D4, Measurement of outcomes; D5, Selection of reported results.

**MINORS items:** ① Study aim ② Patient consecutiveness ③ Prospective data ④ Endpoint appropriateness ⑤ Endpoint objectivity ⑥ Follow-up adequacy ⑦ Loss to follow-up <5% ⑧ Sample size estimation ⑨ Control group appropriateness ⑩ Contemporary control ⑪ Baseline comparability ⑫ Statistical analysis.

**SYRCLE items:** ① Sequence generation ② Baseline adjustment ③ Allocation concealment ④ Animal randomization ⑤ Blinding (performance) ⑥ Random outcome evaluation ⑦ Blinding (detection) ⑧ Incomplete data ⑨ Selective reporting ⑩ Other bias.

**Quality rating criteria:** RoB2 — Low/Some concerns/High; MINORS — Good (≥75%), Moderate (50–74%), Poor (<50%); SYRCLE — Low (0 high-risk), Moderate (0 high but ≥4 unclear), Some concerns (1 high-risk), High (≥2 high-risk).

#### 3.1.1 Study population and sample

**Figure 2:**
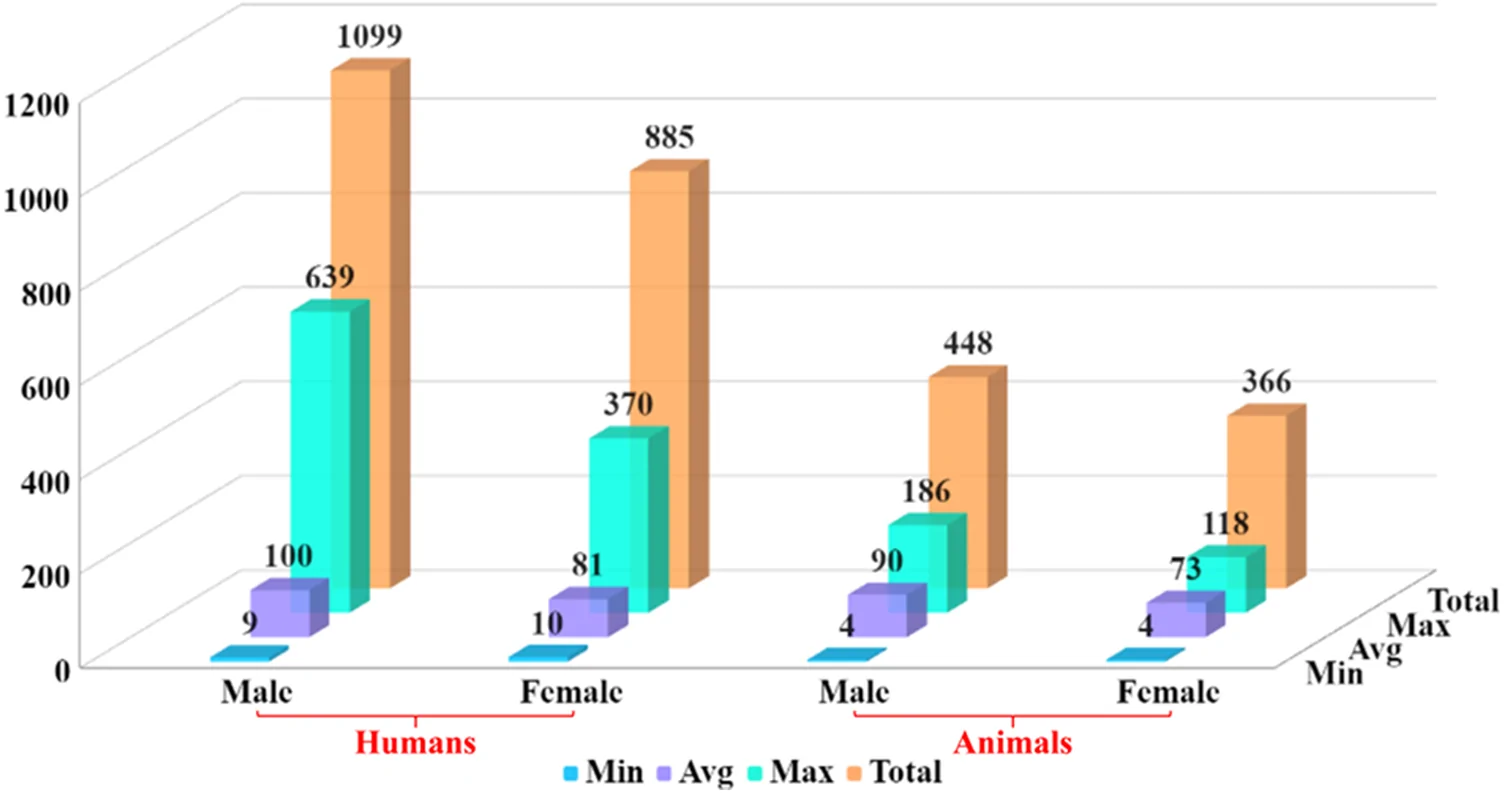
Distribution of sample sizes across included studies.

The total sample size of the included studies was 2,798 cases, including 814 cases of animals and 1,984 cases of humans. In the human studies, the average sample size of male participants was 100 cases (minimum 9 cases, maximum 639 cases), with a total of 1,099 cases; the average sample size of women was 81 cases (minimum 10 cases, maximum 370 cases), with a total of 885 cases. In the animal study, the average sample size of male animals was 90 cases (minimum 4 cases, maximum 186 cases), with a total of 448 cases; the average sample size of female animals was 73 cases (minimum 4 cases, maximum 118 cases), with a total of 366 cases. The distribution of sample sizes and classification statistics is presented in Figure 2.

The human samples included healthy subjects and patients with various diseases, such as severe obesity with hyperlipidemia, anxiety disorder during drug rehabilitation, COVID-19 recovery, chronic low back pain, and depression. Animal samples include castrated SD rats, bone tumor mice, SD rats, C57BL/6 mice, etc. Among them, healthy people, male castrated SD rats, and female castrated SD rats are the most commonly used sample types. The specific sample types are shown in Table 2.

The analysis of the 16 included studies revealed that 93.75% (*n* = 15) reported sex-stratified data. Only 6.25% (*n* = 1) provided qualitative descriptions without quantitative sex-disaggregated data. Regarding the research objectives, half of the studies (50.00%, *n* = 8) identified the analysis of sex differences as their primary focus, centering their design and discussions on exploring sex-specific efficacy and mechanisms of TCM interventions, while 43.75% (*n* = 7) conducted sex-stratified analysis as a secondary, exploratory objective alongside core assessments of intervention effectiveness. For details, see supplementary Appendix 2 and supplementary Appendix 3.

#### 3.1.2 Intervention types

A wide range of TCM interventions was investigated, mostly acupuncture-related studies (*n* = 9), mainly focusing on neural mechanisms, DEQI sensation, and pain management. Single herb studies (*n* = 2) focused on sex-specific pharmacokinetics (e.g., schisandrin) and metabolic efficacy (e.g., honokiol). In the study of TCM compound (*n* = 4), the curative effect of orthopedic diseases (such as osteoporosis) and chronic diseases (such as long-term coronary and stroke sequelae) was observed. In addition, one study explored the effect of non-drug therapies such as Qigong on mental health. The specific intervention methods include single acupuncture, single drug, and combined treatment, such as acupuncture combined with warm acupuncture, acupuncture combined with moxibustion or massage, diet and drug combination, qigong combined with basic treatment, etc., reflecting the characteristics of comprehensive intervention of TCM.

For detailed intervention data statistics, please refer to supplementary Appendix 4.

#### 3.1.3 Controls

The included studies employed different control designs, including inter-group comparisons (comparisons between different interventions), intra-group comparisons (changes before and after the intervention), and, in some cases, subgroup analyses. Regarding acupuncture, longitudinal comparisons were conducted across different acupuncture techniques, and one study additionally evaluated a combined intervention using two types of needles (hand needle plus ciliary needle). Most studies investigating acupuncture as an intervention were controlled clinical trials (CCTs). In contrast, studies using pharmacological interventions were mainly animal experiments and predominantly focused on sex-specific comparisons, adopting inter-group comparisons, intra-group comparisons, or a combination of both for detailed control data statistics. Please refer to supplementary Appendix 4.

#### 3.1.4 Outcomes

The studies assessed sex differences in the efficacy of TCM interventions using a range of multidimensional outcome indicators. The outcome measurements included in the studies covered a wide range of physiological, biochemical, imaging, and psychological parameters. For example, in the osteoporosis study (42), outcomes included bone mineral density, bone metabolism indices, and expression of proteins in related signaling pathways; in the study of pain and the nervous system (32), functional magnetic resonance imaging (fMRI) was used to observe the activation of brain regions, the detection of neurotransmitter concentration and the pain rating scale. In the study of endocrine metabolism (43), the changes in body weight, blood lipid, blood glucose, and hormone levels were evaluated. In the study of mental health (33), anxiety and depression scales and neurotransmitter levels were used as an evaluation basis. For detailed outcomes data statistics, please refer to supplementary Appendix 4.

Although safety represents a relevant dimension when evaluating therapeutic interventions, the present scoping review did not allow for a meaningful assessment of sex-related differences in safety outcomes. None of the included studies explicitly reported adverse events or other safety-related outcomes stratified by sex or gender. Consequently, no conclusions can be drawn regarding the safety profile of the investigated interventions, highlighting an important gap in the current evidence base.

### 3.2 Key findings on sex differences in the efficacy of TCM

Sex-specific differences in the efficacy were observed across various TCM interventions.

#### 3.2.1 Chinese herbal or Chinese patent medicine interventions

With regard to pharmacokinetic profiles, female rats demonstrated significantly higher systemic exposure (AUC_0−t_) and slower elimination (t_1/2_ 2–9 times longer) of schisandra lignans following oral administration of schisandra chinensis extract compared with male rats, indicating pronounced sex-related pharmacokinetics likely mediated by sex-related cytochrome P450 (CYP) enzyme activities (24). The question of whether these pharmacokinetic differences translate into sex-specific clinical outcomes in humans requires further investigation. In fact, in terms of signal pathway regulation, female participants experience more pronounced benefits than male participants, such as the OPG/RANKL/RANK signaling pathway and the Wnt/β-catenin pathway which were modulated by Zuogui Pill and Yougui Pill in osteoporotic models, and the PI3 K/Akt signaling pathway by Yougui Pill and its medicated serum in bone metabolism regulatory pathways (42). Regarding functional improvement, female participants achieved greater benefits in functional outcomes, whereas male participants did not show significant benefits, NeuroAiD (MLC601) is used to improve function in nervous system diseases, and the Bufei Huoxue Capsules are used to enhance lung function and other parameters (27).

#### 3.2.2 Acupuncture interventions

There are sex-based differences in the efficacy of various acupoints and stimulation techniques. Female participants tend to derive greater benefits from acupuncture targeting pain-related acupoints, and male participants experience more pronounced efficacy from acupuncture at acupoints associated with dullness (22). Specifically, female participants show greater response when the GB34 and Xuanzhong acupoint are stimulated, and male participants show greater response when the Hegu acupoint is stimulated. In addition, acupuncture exhibited a combined effect on weight loss and lipid regulation in patients with severe obesity complicated with hyperlipidemia (44). The improvement of weight loss and lipid reduction in male participants is significantly better than that in female participants. However, female participants show superior outcomes in terms of brain network deactivation and overall intervention, such as engage emotion and sensorimotor brain networks, neurotransmitters, and immune cells, and male participants show superior outcomes in terms of sensorimotor networks activation (33).

#### 3.2.3 Combined therapies

In the combination of acupuncture with spinal manipulation therapy, female participants get more benefit from acupuncture, and male participants get more benefit from spinal manipulation therapy. In interventions addressing high-fat diet-induced obesity supplemented with honokiol, male participants experience significant increases in Akkermansia abundance and improvements in obesity-related complications, but female participants do not show notable efficacy. Furthermore, female participants demonstrate more pronounced benefits in TCM interventions for neurotransmitters such as dopamine, 5-hydroxytryptamine, norepinephrine, and *γ*-aminobutyric acid (24).

**Figure 3:**
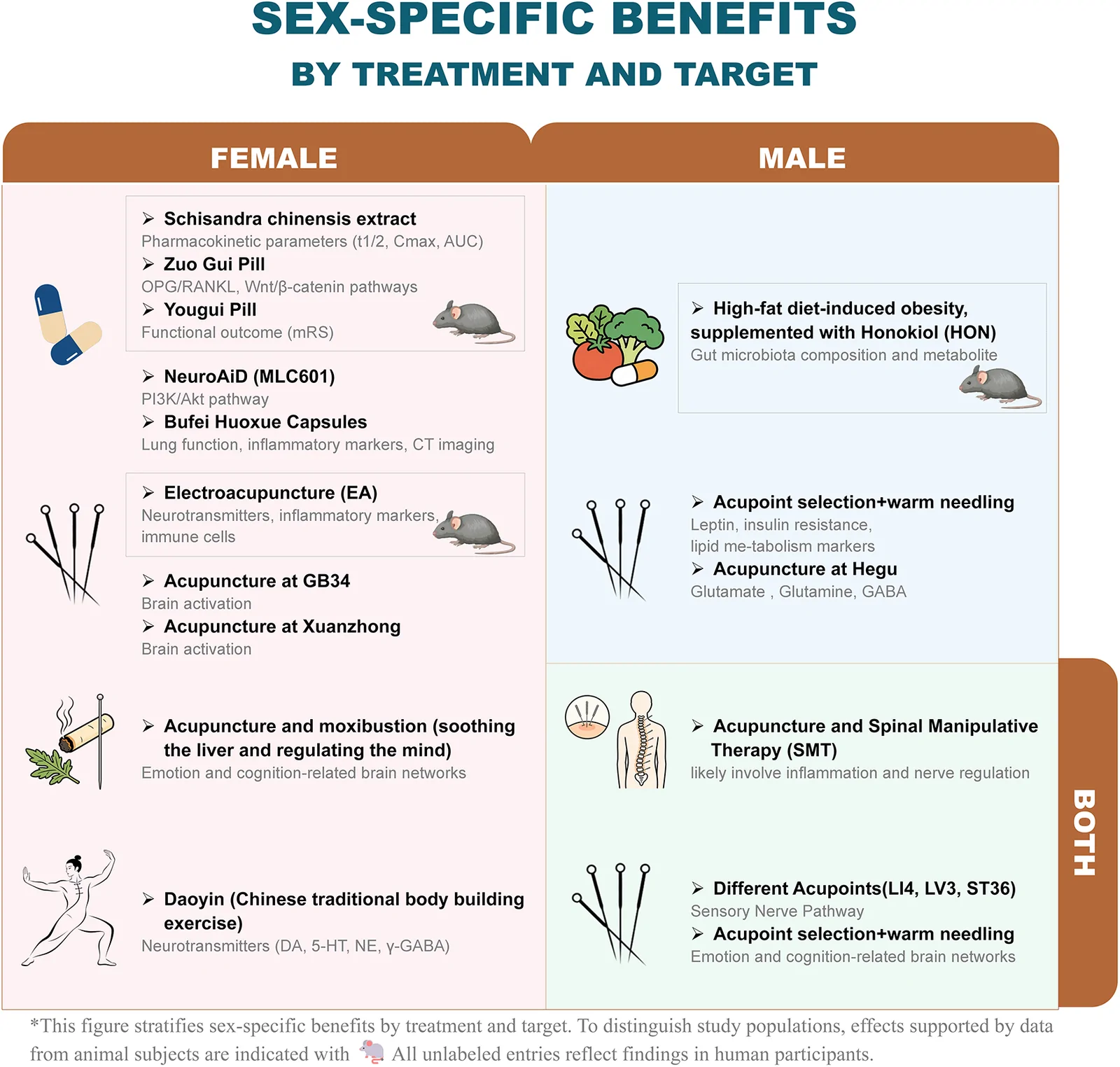
Visual summary of sex-specific benefits of TCM interventions by treatment type and target outcome.

Based on the included literature, a visual chart summarizing the sex-specific benefits by treatment and possible underlying mechanism is shown in Figure 3. The table in supplementary Appendix 7 shows the findings of sex differences in the efficacy of TCM interventions and possible underlying mechanism.

### 3.3 Methodological quality

Regarding the methodological quality of randomized controlled trials, 2 studies were rated as high-risk in terms of “bias in outcome measurement”. The main problems included unclear description of random sequence generation and allocation concealment, insufficient implementation of the blind method, lack of sample size estimation, and no blind method for outcome evaluation. One study was rated as high risk in “results selective reporting bias”. Two items of “overall bias” were rated as high risk. SYRCLE was used to evaluate the methodological quality of 5 animal experiments. In animal experiments, the overall methodological quality was good. However, one study was rated as high risk in the 2 items of “sequence generation” and “random result evaluation” in methodological quality. Non-randomized controlled trials used MINORS for methodological quality evaluation. The evaluation results showed that the items “objectivity of endpoint evaluation” and “sample size estimation” were both rated as high risk. The SAs studies used MINORS for methodological quality evaluation. The evaluation results showed that the items “objectivity of endpoint evaluation” and “sample size estimation” were both rated as high risk. The specific methodological quality results are presented in supplementary Appendix 5.

### 3.4 Quality of reporting

#### 3.4.1 Randomized controlled trials

Regarding the Quality of Reporting of randomized controlled trials, among the 37 main and secondary items, 1a items can be identified as random trials, important changes to the test method after the start of the 3b test, whether the outcome indicators have changed and the reasons after the start of the 6b test, 7b mid-term analysis and test suspension explanation, 12b additional method analysis, 14b test interruption or stop reason explanation, 18 other additional analysis methods results, such as subgroup and correction analysis, 19 test serious harm or unexpected result analysis, the above 8 items were not reported in all RCTs, and the entry specification compliance rate was less than 80%. ARRIVE was used to evaluate the reporting standards of 5 AEs. The animal experiment report was generally good, but 2 studies were rated as high risk in the three items of “randomized use”, “ethical approval”, and “animal care and monitoring”. Non-randomized controlled trials and single-arm studies were evaluated using TREND. The evaluation results showed that the sample size of item 7, 8 allocation method, 9 blind method, 10 analysis units, 18 auxiliary analysis, and 19 adverse reactions were not reported. The specific reporting standards are provided in supplementary Appendix 6.

## 4 Discussion

The scoping review indicates that the volume of publications on the topic remained low from 2009 to 2025, suggesting that research attention has been limited. Furthermore, the limited number of randomised controlled trials and the relatively high proportion of preclinical and non-randomised studies highlight the early stage of research in this area. Notably, all included studies failed to systematically collect or report gender-related variables or conduct gender-sensitive assessments, representing a significant gap in the existing evidence base. This finding suggests a need for more rigorous clinical trials to better evaluate sex and gender differences in the efficacy and safety of TCM interventions.

### 4.1 Potential mechanisms underlying sex differences in the efficacy of TCM

The above studies suggest that the efficacy of TCM varies among male and female patients, indicating the presence of sex-related differences. These differences may arise from a range of underlying biological mechanisms that, based on the existing evidence, can be organized into three key aspects. This part is strictly limited to mechanisms explicitly reported or directly evidenced in the included studies and closely related TCM literature; no extrapolation to unreported mechanisms is attempted. The mechanisms addressed in this review include sex hormone regulation, gut microbiota dimorphism, and nervous system structural and functional differences.

Firstly, differences in the regulation and activity of sex hormones play a central role (45, 46). Sex hormones are among the key biological factors underlying sex-specific differences between male participants and female participants, directly influencing the expression and sensitivity of drug targets. Taking osteoporosis as an example, the sudden decrease in estrogen levels in postmenopausal female participants is the main cause of bone loss, while the kidney-tonifying Chinese medicine Yougui Pill may exert more significant bone-protective effects in female models by simulating or regulating estrogen receptor-related pathways (such as PI3 K/Akt) (30). Similarly, when acupuncture is used for emotional regulation, its effects may be related to the influence of female sex hormones (particularly estrogen) fluctuations on central neurotransmitter levels (43). Sex hormone differences provide an important endocrine basis for the sex-specificity of TCM efficacy.

Secondly, the sex-based dimorphism of gut microbiota plays an important role (47). Often referred to as the “second genome” of the human body, the gut microbiota exhibits significant sex-specific differences in both composition and function. These differences vary across different stages of life and can profoundly influence drug metabolism and immune responses. For example, in a study (29) investigating magnolol's effects on obesity, male models exhibited more efficient metabolic conversion of the drug by their gut microbiota, leading to increased abundance of beneficial bacteria and improved obesity-related indicators, whereas no significant changes were observed in female models. This suggests that the male gut microbiome may be more sensitive to certain Chinese medicine components, leading to sex-specific differences in therapeutic efficacy. Therefore, the sex-specificity of gut microbiota may be one of the key factors mediating differences in the efficacy of TCM.

Thirdly, the structure and function of the nervous system differ between sexes (48). Numerous fMRI studies on acupuncture have demonstrated that male and female brains vary in structure, such as brain volume, gray matter ratio, and functional network connections, leading to distinct neural processing of the same stimuli. Female participants often activate emotion- and cognition-related brain regions during acupuncture, while male participants activate areas like the sensorimotor cortex. This may explain why female participants are more sensitive to mood regulation through acupuncture, whereas male participants experience greater improvements in somatic symptoms (26). Additionally, sex differences in neurotransmitter sensitivity, such as responses to GABA and glutamate, may underlie variations in “DEQI” sensation and analgesic effects (22).

These mechanisms do not function independently; rather, they are interconnected. For example, hormone levels can influence the composition of the intestinal microbiota, and the metabolites produced by the microbiota can impact the nervous system. Concurrently, sex-specific variations in the neuroendocrine system can affect drug absorption, metabolism, and target efficacy. Consequently, the sex-specific efficacy of TCM is likely attributable to a complex interplay of multiple factors, necessitating a comprehensive, holistic approach for study.

### 4.2 Conceptual alignment between “sex and gender medicine” and TCM

Recent evidence on sex-specific differences in TCM efficacy aligns closely with core TCM theoretical principles. Constitution theory in TCM holds that health status and disease susceptibility depend on relatively stable individual characteristics shaped by yin–yang balance, qi and blood, and organ (Zang-fu) function, with sex representing a key determinant of constitutional variation. Modern findings on sex differences in hormones, gut microbiota, and neural regulation may therefore reflect the biological substrates of the TCM concept of constitution, helping to explain sex-related variability in disease risk and treatment response (49).

Moreover, the TCM principle of “treating the same disease with different methods” emphasizes individualized therapy based on patient-specific characteristics. This is supported by evidence showing sex-differentiated responses to interventions such as acupuncture and massage in chronic low back pain, underscoring the relevance of sex-informed therapeutic choices (28).

Finally, the concept of “treating different diseases with the same method” is illustrated by the effectiveness of TCM in conditions such as osteoporosis and emotional disorders in women (42), which may share common biological pathways, including estrogen-regulated neuroendocrine mechanisms.

Overall, Gender Medicine provides a modern scientific framework that strengthens and refines TCM theory, with both approaches converging on the importance of personalized, sex-specific healthcare. This alignment also echoes the growing call for the scientization and evidence-based integration of TCM with modern biomedicine, which lays a foundational basis for translating sex-specific TCM findings into clinical practice (50).

### 4.3 Terminological considerations: “sex” and “gender” in TCM research

We observed inconsistent terminology across the 16 included studies. The slash formulation “sex/gender”, as articulated by Fausto-Sterling (51), conceptualizes sex and gender as irreducibly entangled biological-sociocultural phenomena. However, we think that explicit terminological differentiation can provide greater analytical clarity for current TCM research.

We propose a framework: (1) Biological mechanisms (pharmacokinetics, microbiota, bone metabolism, neuroendocrinology): use “sex-related” or “sex-specific”; (2) Sociocultural outcomes (healthcare behaviors, adherence, patient experiences): use “gender-related” or “gender-associated”; (3) Mixed or undifferentiated domains: use “sex- and gender-related” or other expression based on specific research needs.

This precision is particularly valuable for TCM, given the field's emphasis on holistic assessment and individualized treatment. Clear terminology will enable future studies to disentangle biological sex differences from sociocultural gender influences, advancing more rigorous, equitable gender-aware TCM practice.

### 4.4 Implications for TCM precision using and pharmaceutical development

This study revealed that marketed Chinese patent medicines (Bufei Huoxue Capsules, Naomaitai Capsules-NeuroAiD, Zuogui Pill, and Yougui Pill) and herbal active components (Schisandra chinensis ethanol extract, honokiol) possess significant sex differences in clinical efficacy, pharmacological activity, and pharmacokinetics. These findings not only provide evidence for TCM precision clinical application, but also provide critical reference for herbal active components-based sex-specific pharmaceutical development. This work is expected to promote the industry interest and investment in sex-specific research of TCM.

## 5 Research limitations

This study has several limitations that should be acknowledged, along with their potential implications for interpreting the findings. First, clinical trial registries, narrative reviews, and grey literature (publications whose titles are identified through database searching but whose full texts are not publicly retrievable) were not included in the search strategy, which may have restricted the scope and comprehensiveness of the evidence. Second, the inclusion of studies was limited to publications in Chinese and English, potentially introducing selection bias and limiting the generalizability of the findings. Third, since the included references did not report safety outcomes, this scoping review was unable to perform a systematic assessment of safety-specific profiles. This represents an important gap in the current evidence base, as safety considerations are integral to clinical decision-making. Fourth, in included clinical trials, the average sample sizes (approximately 80–100 cases per sex) are relatively small, which may not detect subtle sex-treatment interaction effects. The conclusions of relevant researches require further validation through more researches in the future. This scoping review does not aim to establish definitive evidence; rather, it seeks to systematically map the current state of research, highlight critical gaps, and call attention to the need for larger, more rigorously designed studies to validate and extend these findings.

## 6 Conclusion

This scoping review systematically assesses the current state of sex- and gender-specific research in TCM. It reveals limited evidence on sex-related differences in TCM efficacy and application, and a notable research gap in the investigation of sex- and gender-specific safety profiles of TCM interventions. Among the included studies, only a minority designated sex difference analysis as a primary objective, while most treated it as an exploratory secondary objective, and a small number lacked sex-stratified data entirely. These findings reflect inadequate integration of sex-related perspectives in TCM research design, highlighting critical research gap and the need for rigorous sex-stratified a clinical trials.

Despite a weak evidence base and suboptimal methodological quality, this review identifies significant sex-specific differences in TCM clinical efficacy. These findings hold important implications for advancing precision medicine in TCM. Clinicians should integrate sex as a key factor in decision-making-alongside the core TCM principle of syndrome differentiation and treatment-to develop tailored interventions that enhance personalized care and therapeutic outcomes (52).

## 7 Implications for research

Future research should prioritize sex- and gender-stratified preclinical and clinical studies, utilizing existing methodological tools to design research protocols that explicitly account for sex- and gender-specific differences (7). Based on the above research, we offer the following three recommendations:

Firstly, regarding research subjects, we advocate that sex- and gender-based analysis should be recognized as an essential methodological dimension and promoted across all TCM research domains. Within this general framework, we particularly recommend that for diseases where TCM demonstrates clear clinical benefits, focused sex- and gender-stratified data mining and statistical analysis should be prioritized, as these conditions represent areas where sex- and gender-specific differences may have the most significant clinical implications. Research should target efficacy, safety, and mechanism of action, advancing through the iterative “practice-theory-practice” spiral model of TCM.

Secondly, in terms of research methodology, a multi-level evidence integration approach, guided by the 6S evidence model, should be adopted. This integration process comprises two sequential stages. Stage 1 involves accumulating a sufficient volume of high-quality studies at each level of the 6S hierarchy: systematic reviews, clinical practice guidelines, synopses, syntheses, studies, and summaries. Stage 2 involves conducting cross-level evidence synthesis to trace connections between molecular mechanisms, cellular responses, animal experiments, and clinical outcomes, thereby building reasonable sex- and gender-specific evidence chains for specific TCM interventions or disease conditions (53).

Thirdly, methodological rigor must be prioritized across the research continuum, and reporting quality requires targeted enhancement. Consistent with contemporary sex and gender research paradigms, investigators should adopt an integrative analytical approach that examines synergistic interactions between sex/gender and multiple contextual factors rather than treating sex/gender merely as a control variable (54–56). In addition to applying the SAGER guidelines to incorporate sex and gender considerations across all research stages (56), there is an urgent need to develop and implement customized TCM-oriented reporting standards. While the SAGER guidelines provide a valuable general framework, they do not address TCM-specific reporting elements such as pattern differentiation (syndrome differentiation), formula composition and modification, acupuncture point selection, and TCM diagnostic criteria—dimensions where sex- and gender-specific differences may be particularly relevant. As empirical evidence indicates that inconsistent reporting undermines the comparability and validity of TCM sex- and gender-related research (57), these standards should build upon existing TCM reporting guidelines (including the CONSORT Extension for Chinese Herbal Medicine Interventions and the STRICTA guidelines for acupuncture) by integrating explicit, operationalized sex and gender reporting requirements. This integration will systematically enhance the scientific rigor, transparency, and reproducibility of sex- and gender-related TCM research.

Additionally, regarding the integration of gender beyond biological sex, we found that none of the included studies examined gender aspects related to sociocultural factors or gender identity, with their focus limited exclusively to biological sex differences. This represents a significant gap in the current body of research, as sex and gender defined not only by biological characteristics but also by social roles, behaviors, and identity-can substantially influence health outcomes, treatment adherence, and patient experiences. The lack of integration of sociocultural gender dimensions in TCM-related studies restricts the comprehensiveness of the evidence and may limit the applicability of findings across diverse populations. Practical guidance for integrating both dimensions is available in the literature, and researchers are encouraged to apply established methodological frameworks that distinguish between sex and gender in study design, data collection, and analysis (54). Future research should adopt a more inclusive framework that distinguishes between sex and gender, and incorporates both dimensions into study design, data collection, and analysis. Doing so would support a more nuanced understanding of individual variability in response to TCM interventions and contribute to more equitable and personalized healthcare strategies.

Given that sex- and gender-sensitive medicine remains nascent in TCM research, many investigators may lack full familiarity with its conceptual frameworks and standardized reporting conventions. The terminological inconsistencies observed in this review highlight this gap. We therefore advocate that TCM researchers first establish foundational competence in sex- and gender-related conceptualizations, adopt standardized terminological frameworks, and familiarize themselves with reporting guidelines before disseminating findings. This methodological foundation will enhance rigor, comparability, and cumulative knowledge building, accelerating the maturation of sex and gender aware TCM research as a robust field of inquiry.

In addition, the included studies did not clearly report safety outcomes stratified by sex. In the future, we recommend establishing unified reporting standards for studies on sex- and gender-specific differences. Specifically, the core outcomes for sex- and gender-specific differences include effectiveness, safety, and other relevant outcomes. These core outcomes should be thoroughly considered during the study design phase and comprehensively reported in published studies.

## Data Availability

The original contributions presented in the study are included in the article/Supplementary Material, further inquiries can be directed to the corresponding authors.

## Supplementary material

Supplementary Appendix 1 Search strategies for each database.

Supplementary Appendix 2 Proportion of studies with sex-stratified data.

Supplementary Appendix 3 Distribution of studies with sex analysis as primary or secondary objective.

Supplementary Appendix 4 PICO(s) of included studies.

Supplementary Appendix 5 Methodological quality assessment results.

Supplementary Appendix 6 Quality of reporting assessment results.

Supplementary Appendix 7 Findings of sex differences in TCM interventions efficacy and possible underlying mechanisms.
