# Correction: Sex-and gender-specific differences in treatment efficacy and safety of traditional Chinese medicine: a scoping review

**DOI:** 10.3389/fgwh.2026.1952542

**Published:** 2026-09-03

**Authors:** Jing Hu, Boyang Liu, Haiyin Hu, Xiaolei Wu, Paola Matarrese, Massimo D'Archivio, Anna Ruggieri, Elena Ortona, Lin Miao, Junhua Zhang, Alice Josephine Fauci, Zhaochen Ji

**Affiliations:** 1 Tianjin University of Traditional Chinese Medicine, Tianjin, China; 2 Haihe Laboratory of Modern Chinese Medicine, Tianjin, China; 3 Key Laboratory of Evidence-Based Evaluation of Traditional Chinese Medicine, National Medical Products Administration, Tianjin, China; 4 Centre for Gender-Specific Medicine, Italian National Institute of Health, Rome, Italy; 5 Sino-Italian Joint Laboratory of Traditional Chinese Medicine, Italian National Institute of Health, Rome, Italy

**Keywords:** scoping review, sex and gender medicine, sex and gender sensitive medicine, sex- and gender-specific differences, traditional Chinese medicine

Error in figure/table

Wrong content

There was a mistake in figure 2 as published. In the published figure 2, the numerical label on the Total bar for Female Animals was incorrectly marked as 119. All numerical descriptions in the main text are consistent with raw data and remain fully accurate; this labeling error arose during figure generation. The correct value should be 366. The corrected figure 2 appears below.

The authors apologize for this inadvertent oversight and confirm that this minor error does not alter any findings or scientific conclusions of the study.

**Figure 1:**
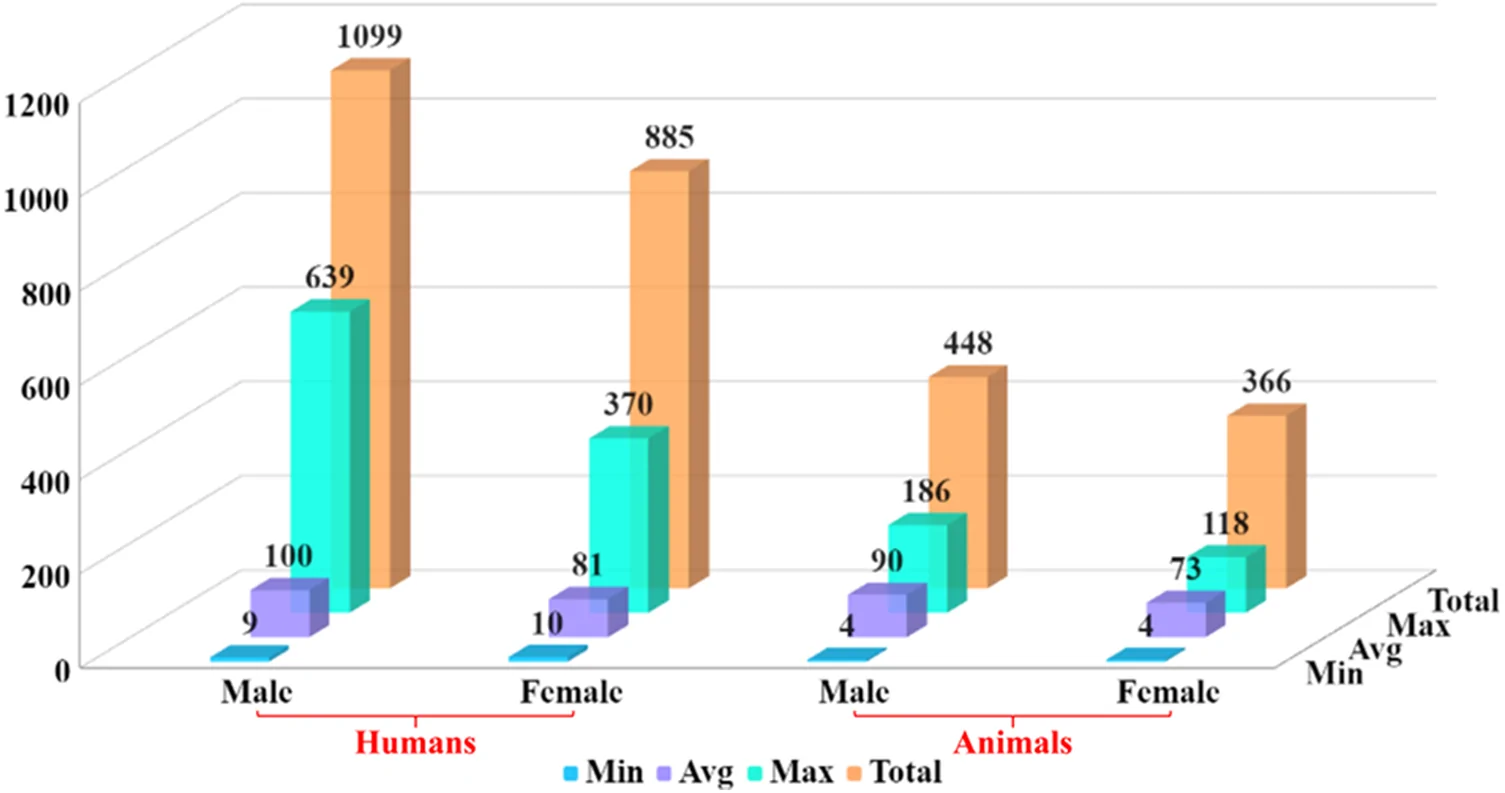


The original version of this article has been updated.

