# Supplementary material for: Sex- and gender-specific differences in treatment efficacy and safety of traditional Chinese medicine: a scoping review
Source: Front Glob Womens Health. 2026 Jul 13;7:1833361. doi: 10.3389/fgwh.2026.1833361 (PMC13402364; doi:10.3389/fgwh.2026.1833361)
Supplement: Supplementary file 1 [file Table1.docx]

Appendix 1. Search Strategies for Each Database

| **Database** | **Database Type** | **Search Field** | **Search Strategy** |
| --- | --- | --- | --- |
| PubMed | English Medical Database | MeSH Terms/Topic | (((((TCM[MeSH Terms]) OR (Traditional Chinese Medicine[MeSH Terms])) OR (Acupuncture[MeSH Terms])) OR (Moxibustion[MeSH Terms])) OR (Chinese Herbal Medicine[MeSH Terms])) AND ((((Gender Medicine[MeSH Terms]) OR (Sex Differences[MeSH Terms])) OR (Male Health[MeSH Terms])) OR (Female Health[MeSH Terms]) OR ((Androgens[MeSH Terms]) OR (Estrogens[MeSH Terms]) OR (Progesterone[MeSH Terms]) OR (Sex Hormones[MeSH Terms]))) |
| CNKI | Chinese Comprehensive Database | Subject (SU) | SU=("Traditional Chinese Medicine" + "Chinese Herbal Medicine" + "Chinese Patent Medicine" + "Integrated Traditional Chinese and Western Medicine" + "Acupuncture" + "Moxibustion") AND SU=("Gender Medicine" + "Sex Differences" + "Male Health" + "Female Health" + "Estrogen" + "Androgen" + "Progesterone" + "Sex Hormones") |
| WanFang (WF) | Chinese Comprehensive Database | Subject (SU) | SU=("Traditional Chinese Medicine" OR "Chinese Herbal Medicine" OR "Chinese Patent Medicine" OR "Integrated Traditional Chinese and Western Medicine" OR "Acupuncture" OR "Moxibustion") AND SU=("Gender Medicine" OR "Sex Differences" OR "Male Health" OR "Female Health" OR "Estrogen" OR "Androgen" OR "Progesterone" OR "Sex Hormones") |
| VIP | Chinese Comprehensive Database | Subject (SU) | SU=("Traditional Chinese Medicine" + "Chinese Herbal Medicine" + "Chinese Patent Medicine" + "Integrated Traditional Chinese and Western Medicine" + "Acupuncture" + "Moxibustion") AND SU=("Gender Medicine" + "Sex Differences" + "Male Health" + "Female Health" + "Estrogen" + "Androgen" + "Progesterone" + "Sex Hormones") |
| SinoMed | Chinese Medical Database | Topic (TI/AB/SU) | (("Traditional Chinese Medicine"[TI/AB/SU] OR "Chinese Herbal Medicine"[TI/AB/SU] OR "Acupuncture"[TI/AB/SU] OR "Moxibustion"[TI/AB/SU] OR "Chinese Patent Medicine"[TI/AB/SU]) AND ("Sex Differences"[TI/AB/SU] OR "Gender Medicine"[TI/AB/SU] OR "Male Health"[TI/AB/SU] OR "Female Health"[TI/AB/SU] OR "Estrogen"[TI/AB/SU] OR "Androgen"[TI/AB/SU] OR "Sex Hormones"[TI/AB/SU])) |
| Web of Science | English Comprehensive Database | Topic (TS) | ("Traditional Chinese Medicine" OR TCM OR Acupuncture OR Moxibustion OR "Chinese Herbal Medicine" OR "Chinese Patent Medicine") AND ("Sex Differences" OR "Gender Medicine" OR "Male Health" OR "Female Health" OR Estrogen* OR Androgen* OR Progesterone OR "Sex Hormones") |

Appendix 2: Proportion of Studies with Sex-Stratified Data

| **Category** | **Number** | **Corresponding Study** |
| --- | --- | --- |
| Studies with sex-stratified data | 15 | Chang Shik Yin 2009^[25]^, Wei Qiao Qiu 2009^[26]^, Branden A. Smeester 2012^[27]^, Haiyan Xu 2013^[28]^, Ling Fan 2015^[30]^, Sujung Yeo 2016^[31]^, Natasha L. Fabiaña 2016^[32]^, Didi Huang 2018^[33]^, Anupama Kizhakkeveettil MAOM 2019^[34]^, Yanan Ding 2019^[35]^, Huihui Xu 2021^[36]^, Yanping Zhao 2023^[37]^, Saiqin Hu 2023^[38]^, Di Miao 2024^[39]^, Chi Hou 2025^[40]^ |
| Studies without sex-stratified data | 1 | Xiaowei Fu 2014^[29]^ |

Appendix 3: Distribution of Studies With Sex Analysis as Primary or Secondary Objective

| **Analysis Objective** | **Number** | **Corresponding Study** |
| --- | --- | --- |
| Sex difference analysis as the primary objective | 8 | N. Venketasubramanian 2010^[26]^, Branden A. Smeester 2012^[27]^, Haiyan Xu 2013^[28]^, Anupama Kizhakkeveettil MAOM 2019^[34]^, Yanan Ding 2019^[35]^, Huihui Xu 2021^[36]^, Yanping Zhao 2023^[37]^, Di Miao 2024^[39]^ |
| Sex difference analysis as a secondary objective | 7 | Chang Shik Yin 2009^[25]^, Ling Fan 2015^[30]^, Sujung Yeo 2016^[31]^, Natasha L. Fabiaña 2016^[32]^,Didi Huang 2018^[33]^, Saiqin Hu 2023^[38]^, Chi Hou 2025^[40]^ |
| No sex-specific assessment | 1 | Xiaowei Fu 2014^[29]^ |

Appendix 4. PICO(s) of Included Studies

| **Reference** | **Objectives** | **Methods** | **Populations/Patients(P)** | **Interventions(I)** | **Comparisons(C)** | **Outcomes(O)** | **Conclusions** | **Limitations** |
| --- | --- | --- | --- | --- | --- | --- | --- | --- |
| Chang Shik Yin 2009^[25]^ | 1. Investigate acupuncture perceptions over 6 points 2. Explore sensory intensity patterns by point and gender | Observational study; factor analysis of sensory questionnaire; cross-gender/point comparison | 121 healthy Korean college students (32F, 89M); acquainted with DEQI, experienced acupuncture | Acupuncture at LI1-LI5, LI11: 0.25×40 mm needles, insertion + 10s alternate rotation + removal | Exp group: 6 acupoint stimulation; no specific control | 1. Females: Higher spreading/numb/transmission perception (P<0.01) 2. Both genders: Higher pain at LI1/LI2, higher dullness at LI4 3. Males: Significant variation in transmission/soreness across points | 1. Acupuncture perception differs by acupoint and gender 2. Four sensory subcategories identified; females score higher in 3 subcategories | 1. Participant bias (familiar with acupuncture theory) 2. No control for participant/practitioner condition variation 3. Results limited to healthy volunteers |
| N. Venketasubramanian 2010^[26]^ | 1. Test sex differences in brain response to acupuncture at LPNN/DMN/SMN 2. Compare neural responses via fMRI | Retrospective fMRI reanalysis; cross-sectional gender comparison; seed-based correlation analysis | 38 healthy right-handed adults (19F,19M); no neuro/psychiatric disorders; 68-74% Caucasian | Manual acupuncture at LI4/LV3/ST36: 180° rotation at 1Hz, 2×2min stimulation in 10min scan | No grouping; 3 acupoint stimulation | 1. Females: Extensive LPNN/DMN deactivation (posterior cingulate/precuneus/angular gyrus, P<0.0001) 2. Males: Stronger sensorimotor cortex activation and connectivity 3. Soreness correlates with deactivation in females only | 1. Gender-specific brain activation patterns exist in acupuncture response 2. Females show greater LPNN/DMN modulation; males show stronger SMN response | 1. No menstrual cycle data for females 2. No formal psychiatric screening 3. Study not originally designed for gender difference analysis |
| Branden A. Smeester 2012^[27]^ | 1. Examine EA for bone tumor pain; test sex differences 2. Investigate EA anti-inflammatory effects | Controlled animal study; 5 EA regimens; von Frey/ELISA/IHC assays; blinded outcome assessment | 270 mice (104F BALB/c,166M BALB/c,20M C3H); tumor-bearing or saline-injected | EA at ST36: 4Hz, 100μs pulse, 30min session; 5 regimens (frequency/timing varied) | 5 EA regimens vs. sham/non-acupoint/tumor-no EA controls | 1. EA-2X/3 most effective: males show earlier relief, females show longer effect 2. EA-2X/3 reduces tumor neutrophil density and PGE2 in males 3. Sham/non-acupoint EA has no effect | 1. EA efficacy depends on regimen; sex differences in response exist 2. EA antihyperalgesic effect linked to anti-inflammatory mechanisms | 1. No test of other EA regimens on inflammation 2. No adsorption controls for IHC 3. Mouse results not directly translatable to humans |
| Haiyan Xu 2013^[28]^ | 1. Investigate gender effects on schisandra lignan pharmacokinetics 2. Compare single vs. multiple dose behaviors | In vivo rat pharmacokinetic study; UPLC-MS/MS for plasma concentration measurement | 8 Sprague-Dawley rats (4F,4M); 200±10g; controlled housing conditions | Schisandra ethanol extract: 20mg/kg oral dose; single vs. twice-daily ×7d multiple dose | Single dose vs. multiple dose groups; gender comparison | 1. Females: 2-9× longer t1/2, 5-50× higher Cmax/AUC0-t vs. males (except schisantherin A Cmax) 2. Multiple doses only affect γ-schisandrin in females | 1. Schisandra lignan pharmacokinetics show significant gender differences 2. Repeated dosing has minimal impact except for γ-schisandrin in females | 1. Schisandrin C concentration below LLOQ; no data 2. Results differ from previous studies (dosage/preparation differences) |
| Xiaowei Fu 2014^[29]^ | 1. Reveal sex differences of Left/Right Gui Wan on osteoporosis 2. Explore "Kidney governs bone" theory mechanisms | Randomized animal study; castrated rat model; double tetracycline labeling for bone histomorphometry | 120 Sprague-Dawley rats (equal F/M); castrated (OVX/ORX) or sham; SPF grade | Left/Right Gui Wan decoction: 20g crude drug/kg, oral once daily (7d on/2d off ×3 months); positive controls: estradiol/methyltestosterone | Blank/sham/model/positive control/Left/Right Gui Wan groups; gender comparison | Qualitative: Left Gui Wan effective in females only; Right Gui Wan effective in both genders; regulates OPG/RANKL and Wnt/β-catenin pathways | 1. Castration induces high-turnover osteoporosis with gender differences 2. Left/Right Gui Wan exhibit sex-specific efficacy via bone regulatory pathways | 1. Short study duration; no full bone mass recovery 2. Mechanisms not fully elucidated, especially in males |
| Ling Fan 2015^[30]^ | 1. Assess acupuncture/moxibustion for depression QoL; test sex differences 2. Compare efficacy between treatment methods | Multicenter single-blind RCT; 3 parallel groups; longitudinal assessment (pre/post/1/3 months) | 143 depression patients (51M,92F); 18-70y; TCM syndrome: liver qi stagnation | Soothing liver acupuncture + moxibustion: LI4/LR3/GV20/GV29 needling; moxibustion at BL17/BL19; intradermal needling at BL15/BL18; twice weekly ×12 weeks | 3 groups: soothing liver therapy vs. acupoint shallow puncture vs. non-acupoint shallow puncture | 1. Soothing liver group: No gender difference in overall efficacy; females more sensitive to SCL90 improvements 2. Shallow puncture group: Males show better HAMD reduction vs. females | 1. Soothing liver therapy has comparable efficacy in both genders 2. Females are more sensitive to the therapy than other methods | 1. SCL90/HAMD are self-reported; prone to bias 2. No control for marriage/living conditions 3. Small sample size limits generalizability |
| Sujung Yeo 2016^[31]^ | 1. Examine gender differences in psychophysical/brain response to GB34 acupuncture 2. Investigate pain matrix neural substrates | Observational study; fMRI BOLD signal analysis; within-subject sham vs. MA comparison | 19 healthy Asian adults (9M,10F); right-handed; no chronic/neuro/psychiatric diseases | Manual acupuncture at GB34: 0.25×40 mm needle, 1cm insertion; 1min rest + 1min 1Hz rotation ×2 cycles | MA vs. sham acupuncture (blunt needle skin touch); gender comparison | 1. Females: Higher aching de qi score; activation in prefrontal/insula/amygdala 2. Males: Activation in thalamus/caudate body 3. Common activation: right cingulate gyrus/caudate body | 1. GB34 acupuncture induces gender-specific brain activation patterns 2. Females show greater medial pain system activation and aching sensation | 1. Small sample size; no confounder stratification 2. No prospective sample size calculation 3. Wide age range may affect results |
| Natasha L. Fabiaña 2016^[32]^ | Investigate sex influence on NeuroAiD efficacy for post-stroke recovery | Post hoc subgroup analysis; multicenter RCT; adjusted statistical analysis | 1009 ischemic stroke patients (639M,370F); CHIMES trial participants | NeuroAiD (MLC601) capsules: 4 capsules ×3 times daily; 3-month intervention | NeuroAiD vs. placebo; gender subgroup comparison | 1. Females: NeuroAiD group has higher good functional outcome rate vs. placebo (p=0.056) 2. Males: No significant difference between NeuroAiD and placebo 3. Sex-treatment interaction trend (p=0.075) | 1. Sex influences NeuroAiD efficacy for post-stroke recovery 2. Females benefit more from NeuroAiD than males | Post hoc analysis; results need further verification |
| Didi Huang 2018^[33]^ | 1. Observe acupuncture for severe obesity/hyperlipidemia 2. Explore gender differences in efficacy | Observational study; pre-post comparison; TCM syndrome differentiation-based treatment | 264 middle-aged patients (106M,158F); severe obesity + hyperlipidemia; 6 TCM syndrome types | Acupuncture + warm needle moxibustion: syndrome-specific acupoints; moxibustion at Qihai/Zusanli etc.; once every other day ×3 months | Acupuncture group vs. conventional treatment group; gender comparison | 1. Total effective rate: 91.5% (M) vs.85.4% (F) (no statistical difference) 2. Males: Greater reductions in weight/BMI/body fat/TC/TG/LDL-C vs. females | 1. Acupuncture has dual weight-loss and lipid-regulating effects 2. Males show superior improvement in obesity and lipid indicators vs. females | No reported limitations |
| Anupama Kizhakkeveettil MAOM 2019^[34]^ | Investigate gender association with acupuncture/SMT efficacy for LBP | Secondary analysis of RCT; 60-day follow-up; regression model for gender-specific outcomes | 80 LBP patients (40M,40F); no baseline differences in age/QoL/pain | 3 treatment groups: acupuncture alone vs. SMT alone vs. acupuncture + SMT; 6-week treatment | 3 treatment groups; gender subgroup comparison | 1. Females: Better pain/disability reduction with acupuncture alone (3.8-point vs.2.0-point pain reduction) 2. Males: Better response with SMT alone (3.5-point vs.1.8-point pain reduction) 3. Combined treatment reduces efficacy in both genders | 1. Gender is associated with treatment response for LBP 2. Females benefit more from acupuncture; males benefit more from SMT | 1. Small sample size; 20% follow-up loss 2. Unblinded participants; self-reported outcomes 3. No Chinese herbs used; no collaborative care model4. No non-binary gender options |
| Yanan Ding 2019^[35]^ | 1. Examine honokiol for HFD-induced obesity; test sex differences 2. Investigate gut microbiota/metabolite mechanisms | Animal experiment; C57BL/6 mice; 5 groups (ND/HFD/HFD+3 HON doses); 8-week intervention | 120 SPF C57BL/6 mice (60F,60M); 6w old; ND/HFD/HFD+200/400/800mg/kg HON | Honokiol supplementation: 200/400/800mg/kg BW; 8-week oral administration with HFD | ND vs. HFD vs. 3 HON dose groups; gender comparison | 1. HON reduces body weight/adipose tissue/serum lipids in both genders; males show earlier weight reduction 2. Males: HON enriches Akkermansia; females: no such effect 3. HON increases SCFAs and decreases LBP in both genders | 1. Honokiol ameliorates HFD obesity via gut microbiota/metabolite modulation 2. Therapeutic effects exhibit significant sex differences | 1. Mechanism of HON needs further verification 2. Animal genetic background may affect gut microbiota results |
| Huihui Xu 2021^[36]^ | 1. Reveal sex differences of Yougui Pill on osteoporosis 2. Clarify "Kidney governs bone" theory via PI3K/Akt pathway | In vivo (rat) + in vitro (cell) experiment; castrated model; PI3K agonist intervention | 156 rats (78F,78M) for in vivo; 80 rats for serum preparation; castrated (OVX/ORX) or sham; SPF grade | Yougui Pill decoction: 10.24g crude drug/kg oral ×13 weeks (in vivo); 20.18g/kg ×7d (serum preparation); PI3K agonist 740Y-P (10μM, 24h) | Blank/sham/model/positive control/Yougui Pill groups; gender comparison; agonist intervention | 1. In vivo: Yougui Pill reduces bone loss in both genders; females show better efficacy (higher BMD/BV/TV, lower SMI) 2. In vitro: Yougui Pill serum inhibits osteoclast differentiation; females show stronger effect 3. Regulates PI3K/Akt pathway in osteoblasts/osteoclasts | 1. Castration induces gender-specific osteoporosis via PI3K/Akt pathway activation 2. Yougui Pill exhibits sex-specific efficacy by inhibiting PI3K/Akt pathway | In vitro experiments use isolated cells; do not reflect in vivo osteoblast-osteoclast interaction |
| Yanping Zhao 2023^[37]^ | 1. Observe Glu/Glx/GABA changes in prefrontal cortex after LI4 stimulation 2. Analyze gender differences in neurotransmitter responses | Prospective observational study; MRS MEGA-PRESS sequence; within-subject pre/during stimulation comparison | 68 healthy volunteers (19M,49F); 20-30y; right-handed; no MRI contraindications | Manual acupuncture at LI4: 0.40×25mm needle, 10-26mm insertion; 30s twirling ×3 cycles; von Frey filament stimulation as control | Manual acupuncture vs. von Frey stimulation; gender comparison; pre/during stimulation comparison | 1. Males: Higher Glu⁺/Glx⁺ concentrations pre/during stimulation vs. females (P<0.05) 2. No gender difference in GABA⁺ concentration 3. Acupuncture has minimal effect on neurotransmitter levels in both genders | 1. Excitatory neurotransmitter concentrations show gender differences in prefrontal cortex 2. LI4 acupuncture has gender-specific effects on Glu/Glx levels | 1. Unequal M/F sample size; potential bias 2. Narrow age range; no menstrual cycle control for females 3. No plasma hormone measurement; no disease state comparison |
| Saiqin Hu 2023^[38]^ | 1. Investigate gender differences in brain response to Xuanzhong acupuncture 2. Compare task-state fMRI activation and resting-state ReHo values | Observational study; task-state/resting-state fMRI; DPARSFA/SPM12 data analysis | 25 healthy adults (13M,12F); 25-32y; right-handed; no MRI contraindications; females not in menstruation/pregnancy | Acupuncture at Xuanzhong: 0.5-1 inch insertion; 180°±20° twirling (60-90 times/min) until DEQI; block design (1min twirling/1min retention ×3 cycles) | No acupuncture control; gender comparison; task-state vs. resting-state comparison | 1. Task-state: Females activate orbital frontal/posterior central gyrus; males activate cerebellum/occipital gyrus 2. Resting-state: Males show enhanced ReHo in calcarine cortex; reduced ReHo in cerebellum/hippocampus vs. females | 1. Xuanzhong acupuncture induces gender-specific brain activation patterns 2. Brain functional changes differ by gender in both task and resting states | 1. Small sample size; no randomized controlled design 2. Only healthy participants; no patient group 3. No follow-up evaluation of cumulative effects |
| Di Miao 2024^[39]^ | 1. Observe gender differences in TCM guided therapy for post-addiction anxiety 2. Investigate neurotransmitter mechanisms | Randomized controlled trial; 2 groups; 3-month intervention; HAMA scale + neurotransmitter measurement | 80 post-addiction anxiety patients (40M,40F); mild-moderate anxiety; morphine urine test negative | TCM guided therapy: daily 1h self-massage (Baihui/Fengchi/Yintang etc.) + Qigong; 3-month duration; routine detoxification for both groups | Male control group (routine detoxification) vs. female treatment group (TCM therapy + routine detoxification) | 1. Total effective rate: 87.5% (F) vs.65.0% (M) (P<0.05) 2. Females: Greater reduction in HAMA total/somatic/mental scores vs. Males 3. Females: Higher increases in serum 5-HT/DA/NE/γ-GABA vs. males | 1. TCM guided therapy effectively improves post-addiction anxiety 2. Female patients show superior efficacy and neurotransmitter response vs. males | 1. Small sample size; no long-term follow-up 2. Only mild-moderate anxiety patients; no severe cases 3. No male treatment group; direct gender comparison limited |
| Chi Hou 2025^[40]^ | 1. Determine BFHX efficacy in post-COVID-19 convalescent subgroups 2. Identify sex/age/severity/antibody effects on BFHX efficacy | Post hoc subgroup analysis; multicenter double-blind RCT; 3-month follow-up | 129 post-COVID-19 patients (BFHX: 64, placebo:65; 48.44%M in BFHX, 44.62%M in placebo); Han nationality; mild/severe acute illness | Bufei Huoxue Capsules (BFHX): oral administration for 3 months | BFHX vs. placebo; subgroup analysis by sex/age/severity/antibody status | 1. Females: BFHX group shows better 6MWD/FAI improvement vs. placebo (P<0.05) 2. Males: BFHX group has decreased chest CT mean CT value vs. placebo (P<0.05)3. BFHX benefits young/mild/antibody-positive/negative subgroups | 1. BFHX improves clinical symptoms and lung recovery in post-COVID-19 patients 2. Females/young/mild patients benefit more from BFHX | 1. Small sample size; limited generalizability 2. Severe case sample small; follow-up loss potential 3. No pulmonary function tests; post hoc analysis has confounding factors |

Appendix 5: Methodological Quality Results

| RCTs |  |
| --- | --- |
| AEs |  |
| CCTs |  |
| SAs |  |

Appendix 6: Methodological Quality Results

| RCTs |  |
| --- | --- |
| AEs |  |
| CCTs and SAs |  |

Appendix 7: Findings of sex differences in TCM interventions efficacy and possible underlying mechanism

| **Group** | **Intervention Type** | **Beneficial Indicators (Sex-Specific)** | **Corresponding Study** |
| --- | --- | --- | --- |
| Females | Acupuncture | Pain perception ratings (spreading/numb/transmission) | Chang Shik Yin 2009^[25]^, N. Venketasubramanian 2010^[26]^, Sujung Yeo 2016^[31]^, Anupama Kizhakkeveettil MAOM 2019^[34]^, Chi Hou 2025^[40]^ |
|  |  | Brain deactivation in posterior cingulate/precuneus |  |
|  |  | "Aching" de qi subscore |  |
|  |  | Brain activation in medial pain system |  |
|  |  | Low back pain/disability improvement (acupuncture alone) |  |
|  |  | Post-COVID-19 6MWD/FAI improvement |  |
|  | Drugs | Depression symptom reduction (soothing liver group) | Ling Fan 2015^[30]^, Huihui Xu 2021^[36]^, Di Miao 2024^[39]^ |
|  |  | Anxiety efficacy (higher total effective rate + neurotransmitter increases) |  |
|  |  | Less bone loss (Yougui Pill) |  |
|  |  | Lower osteoclast activity (TRAP/RANKL) |  |
|  | Pharmacology | Longer t1/2 & higher Cmax/AUC0-t (Schisandra lignans) | Haiyan Xu 2013^[28]^, Yanan Ding 2019^[35]^ |
|  |  | Weight reduction (HON, 7-week onset) |  |
| Males | Acupuncture | Soreness rating variation | Chang Shik Yin 2009^[25]^, N. Venketasubramanian 2010^[26]^, Anupama Kizhakkeveettil MAOM 2019^[34]^ |
|  |  | Brain activation in left angular gyrus |  |
|  |  | Sensorimotor network correlations |  |
|  |  | Low back pain improvement (SMT alone) |  |
|  | Drugs | Obesity-related indicator reduction (weight/BMI/blood lipids) | Ling Fan 2015^[30]^, Didi Huang 2018^[33]^, Chi Hou 2025^[40]^ |
|  |  | Depression symptom reduction (acupoint shallow puncture group) |  |
|  |  | Post-COVID-19 chest CT mean CT value decrease |  |
|  | Pharmacology/Mechanism | Earlier hyperalgesia relief (EA-2X/3) | Branden A. Smeester 2012^[27]^, Yanan Ding 2019^[35]^ |
|  |  | Reduced tumor neutrophil density/PGE2 |  |
|  |  | Gut microbiota enrichment (Akkermansia via HON) |  |
|  |  | Weight reduction (HON, 4-week onset) |  |
